# *Notes from the Field*: Parvovirus B19 Activity — United States, January 2024–May 2025

**DOI:** 10.15585/mmwr.mm7423a3

**Published:** 2025-06-26

**Authors:** Alfonso C. Hernandez-Romieu, Kelly Carey, Stephanie Dietz, Aaron Kite-Powell, Olivia Almendares, Hannah L. Kirking

**Affiliations:** ^1^Coronavirus and Other Respiratory Viruses Division, National Center for Immunization and Respiratory Diseases, CDC; ^2^Detect and Monitor Division, Office of Public Health Data, Surveillance, and Technology, CDC.

**Summary**

**What is already known about this topic?**

Parvovirus B19 (B19) is a respiratory virus that can cause adverse fetal outcomes in pregnant women and persons who are immunocompromised or have chronic hemolytic blood disorders. After relatively low rates during the COVID-19 pandemic years of 2021–2023, B19 activity in 2024 exceeded that of prepandemic years.

**What is added by this report?**

Data from the National Syndromic Surveillance Program indicated that the proportion of sera specimens positive for B19 antibodies during January–May 10, 2025, was higher than during the same period in 2024, suggesting a sustained increase in B19 transmission.

**What are the implications for public health practice?**

Health care providers should have a heightened suspicion of and consider providing testing for B19 infection among groups at high risk for severe outcomes, including pregnant women with compatible symptoms or exposure to B19. Among pregnant women, health care providers should remain vigilant for fetal complications related to B19 infection. Pregnant women and persons at increased risk for complications from B19 infection might consider using additional prevention strategies (e.g., wearing a mask around other persons).

Parvovirus B19 (B19) is a respiratory virus primarily transmitted through the air by persons with symptomatic or asymptomatic infection. B19 infection causes mild illness in most persons but can result in adverse fetal outcomes in pregnant women or severe disease in persons who are immunocompromised or have chronic hemolytic blood disorders. No antiviral medication exists to treat B19 infection. B19 activity typically peaks in the second quarter of the year (April–June). After low rates during the COVID-19 pandemic (2021–2023), B19 activity in 2024 exceeded prepandemic years, and CDC released a Health Advisory in August 2024 (*1*,*2*).

## Investigation and Outcomes

To determine whether increased B19 activity continued from 2024 into 2025, CDC analyzed data on serum B19-specific immunoglobulin M (IgM) antibodies, a marker of recent infection. Data were obtained from CDC’s National Syndromic Surveillance Program (NSSP)* and originated from tests conducted by a large commercial laboratory. IgM antibodies were assayed using an enzyme immunoassay approved by the Food and Drug Administration; an index value >1.1 indicates antibody detection. Laboratory test data were consistently received from all 10 U.S. Department of Health and Human Services’ regions^†^ during the study period. Region 2 (New Jersey, New York, Puerto Rico, and the U.S. Virgin Islands) was overrepresented compared with the U.S. population.

The weekly number and proportion of positive IgM test results among children and adults submitted to NSSP during January 1, 2023–May 10, 2025, were summarized by week. Positivity ratios (PR) were calculated by dividing the proportion of positive IgM test results in the first two quarters of 2025 by the proportion of positive IgM test results in the same quarters of 2024. PRs with 95% CIs were examined overall and by age group; 95% CIs that excluded the value of 1.0 were considered statistically significant. This activity was reviewed by CDC, deemed not research, and was conducted consistent with applicable federal law and CDC policy.^§^

In 2024, the proportion of positive IgM test results increased from 3.3% during mid-February (week 7) to a peak of 9.6% in late June (week 27) and then decreased to a low of 2.0% during late October (week 44) (Figure). The proportion of positive IgM test results increased from 2.8% in mid-November 2024 (week 46) to 7.3% in early May 2025 (week 19). The number of weekly IgM tests performed in 2025 (mean = 1,401; 95% CI = 1,333–1,469) was similar to 2024 (mean = 1,328; 95% CI = 1,278–1,377). The proportion of tests ordered for females and males was almost equal for all age groups except 15–44 years (93% female) and ≥45 years (66% female).

**FIGURE:**
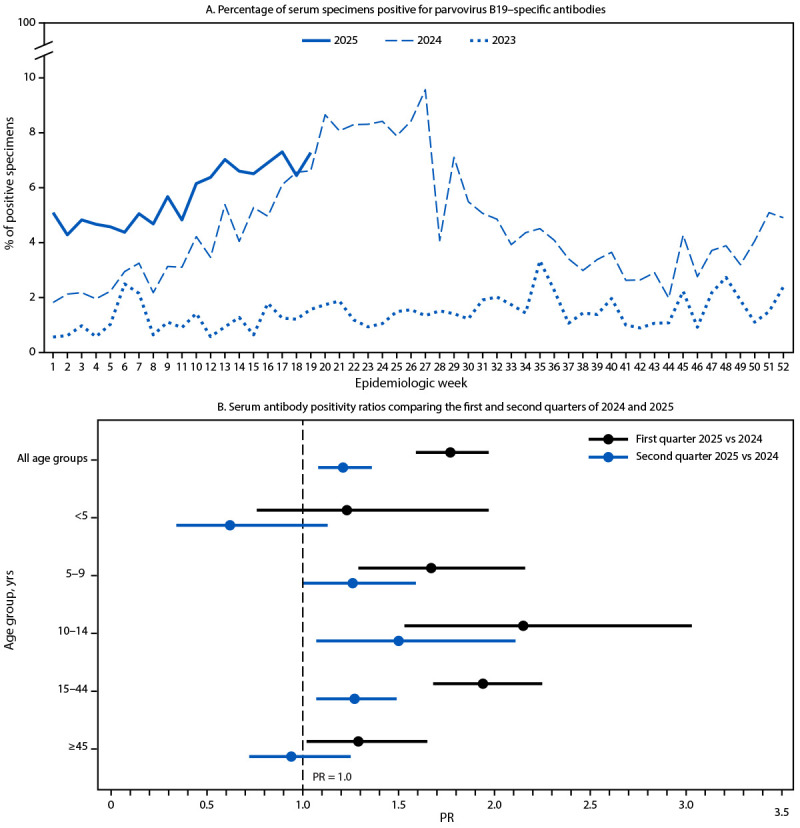
Percentage of serum specimens positive for parvovirus B19–specific antibodies,* by epidemiologic week (A), and serum antibody positivity ratios^†^ comparing the first and second quarters of 2024 and 2025,^§^ by age group (B) — National Syndromic Surveillance Program,^¶^ 2023–2025 **Abbreviations:** IgM = immunoglobulin M; PR = positivity ratio. * IgM antibodies. ^†^ PR is the percentage of all IgM antibody–positive test results during a specified period divided by the percentage of all IgM antibody–positive test results during the comparison period. PRs with 95% CIs were examined overall and by age group; 95% CIs that excluded 1.0 were considered statistically significant. ^§^ With 95% CIs indicated by bars. ^¶^ The National Syndromic Surveillance Program is a collaboration among CDC, local and state health departments, and federal, academic, and private sector partners. National Syndromic Surveillance Program | CDC

Compared with 2024, the proportion of positive IgM test results in 2025 was significantly higher in both the first (PR = 1.8; 95% CI = 1.6–2.0) and second quarter (PR = 1.2; 95% CI = 1.1–1.4) (Figure). Except for children aged <5 years, PRs of all age groups were significantly higher in the first quarter of 2025 than in 2024, with the highest estimate in children and adolescents aged 10–14 years (PR = 2.2; 95% CI = 1.5–3.0). In the second quarter, PRs among those aged 10–14 years and 15–44 years were significantly higher in 2025 than in 2024.

## Preliminary Conclusions and Actions

B19 IgM test data for persons receiving testing through May 10, 2025, indicate increased, sustained B19 transmission, particularly among persons aged 10–14 and 15–44 years, which includes women of reproductive age. These estimates might be an undercount because they relied on clinician testing; parvovirus B19 infection, especially mild infection, is likely far more prevalent than represented in these data. Early identification of B19 infection can prompt early detection and treatment of severe anemia, helping to reduce adverse fetal outcomes and severe disease in persons who are immunocompromised or have chronic hemolytic blood disorders. For this reason, health care providers should consider testing 1) pregnant women who might have been exposed to B19 and 2) persons at increased risk for severe disease and who have signs and symptoms including fever, rash, arthropathy, or unexplained anemia with low reticulocyte count (*3*). Health care providers caring for pregnant patients should remain vigilant for signs of reduced fetal movement or evidence of hydrops, which could be associated with B19 infection (*4*,*5*). Pregnant women and persons at increased risk for complications from B19 infection might consider using additional prevention strategies, such as wearing a mask around other persons.

## Footnotes

*
National Syndromic Surveillance Program | CDC

†
Regional Offices | U.S. Department of Health and Human Services

§ 45 C.F.R. part 46.102(l)(2), 21 C.F.R. part 56; 42 U.S.C. Sect. 241(d); 5 U.S.C. Sect. 552a; 44 U.S.C. Sect. 3501 et seq.

## References

[1] Alfego D, Hernandez-Romieu AC, Briggs-Hagen M, Detection of increased activity of human parvovirus B19 using commercial laboratory testing of clinical samples and source plasma donor pools—United States, 2024. MMWR Morb Mortal Wkly Rep 2024;73:1076–81. 10.15585/mmwr.mm7347a2 39602409 PMC11602021

[2] CDC. Increase in human parvovirus B19 activity in the United States. Atlanta, GA: US Department of Health and Human Services, CDC; 2024. https://www.cdc.gov/han/2024/han00514.html

[3] Young NS, Brown KE. Parvovirus B19. N Engl J Med 2004;350:586–97. 10.1056/NEJMra030840 14762186

[4] Nordholm AC, Trier Møller F, Fischer Ravn S, Epidemic of parvovirus B19 and disease severity in pregnant people, Denmark, January to March 2024. Euro Surveill 2024;29:2400299. 10.2807/1560-7917.ES.2024.29.24.2400299 38873795 PMC11177569

[5] Russcher A, Verweij EJ, Maurice P, Extreme upsurge of parvovirus B19 resulting in severe fetal morbidity and mortality. Lancet Infect Dis 2024;24:e475–6. 10.1016/S1473-3099(24)00373-6 38901439

